# Correlates of self-reported offending in children with a first police contact from distinct socio-demographic and ethnic groups

**DOI:** 10.1186/1753-2000-5-22

**Published:** 2011-06-29

**Authors:** Lieke van Domburgh, Theo AH Doreleijers, Charlotte Geluk, Robert Vermeiren

**Affiliations:** 1 VU University Medical Center, Department of Child and Adolescent Psychiatry, PO BOX 303, 115 ZG Duivendrecht, The Netherlands; 2 LSG-Rentray, PO BOX 94, 7200 AB Zutphen, The Netherlands; 3 Curium-LUMC, Leiden University Medical Center, Department of Child and Adolescent Psychiatry, PO BOX 15, 2300 AA Leiden, The Netherlands; 4 Leiden University, Law Faculty, PO BOX 9520, 2300 RA Leiden, The Netherlands

## Abstract

**Background:**

This study aims to identify risk factors for level of offending among childhood offenders from different socio-economic status (SES) neighborhoods and ethnic origins.

**Method:**

Three groups of childhood first time police arrestees were studied using standardized instruments for individual and parental characteristics: native Dutch offenders from moderate to high SES neighborhoods, native Dutch offenders from low SES neighborhoods, and offenders of non-Western origin from low SES neighborhoods.

**Results:**

All subgroups showed high rates of externalizing disorders (27.2% to 41.8%) and familial difficulties (25.7% to 50.5%). Few differences between neighborhoods were found in the prevalence and impact of risk factors. However, the impact of some family risk factors on offending seemed stronger in the low SES groups. Regarding ethnical differences, family risk factors were more prevalent among non-Western childhood offenders. However, the association of these factors with level of offending seemed lower in the non-Western low SES group, while the association of some individual risk factors were stronger in the non-Western low SES group. Turning to the independent correlation of risk factors within each of the groups, in the Dutch moderate to high SES group, 23.1% of the variance in level of offending was explained by ADHD and behavioral problems; in the Dutch low SES group, 29.0% of the variance was explained by behavioral problems and proactive aggression; and in the non-Western low SES group, 41.2% of the variance was explained by substance use, sensation seeking, behavioral peer problems, and parental mental health problems.

**Conclusions:**

Thereby, the study indicates few neighborhood differences in the impact of individual and parental risk factors on offending, while individual and parental risk factors may differ between ethnic groups.

## Background

Inconsistency surrounds the issue of the impact of risk factors on juvenile offending in affluent versus disadvantaged neighborhoods [1,2]. Some argue that juveniles in disadvantaged neighborhoods are marked by more but not different risk factors, while others have found no differences in risk factors, but found the impact of certain risk factors on offending to be stronger among juveniles who reside in disadvantaged neighborhoods [for a review see [1]]. As most studies on juvenile offending included samples from disadvantaged neighborhoods only, empirical studies on this issue are limited.

The issue is further complicated as neighborhoods of different socio-economic status (SES) also tend to differ in other population characteristics. For instance, minority groups are overrepresented in disadvantaged as compared to better-off neighborhoods [3]. As a result, it becomes difficult to conclude whether reported differences in risk factors between juveniles residing in different neighborhoods can be attributed to differences in SES or to differences in ethnic background. Similarly, studies on the influence of ethnic background on offending have been inconsistent and due to the overrepresentation of minorities in low SES neighborhoods, most studies have not been able to rule out the influence of SES [1,3]. Therefore, the role of ethnic background cannot be ignored when studying the influence of neighborhood SES on risk factors for offending.

In addition, the impact of neighborhood SES has only been studied in general population studies, while no studies have focused on the impact of risk factors on the level of offending among children who have already committed an offense. Focusing on offending in younger children, defined as committing a first offense before puberty, may bear specific relevance, as childhood onset offenders, particularly those whose offending behavior has resulted in an early official police arrest, have a higher risk of becoming serious and persistent offenders when compared to adolescent onset offenders [4-6]. Therefore, this study aims to identify risk factors for frequency of (self and parent reported) offending in childhood first-time police arrestees^1 ^from different neighborhoods, taking into account ethnic origin.

### SES and offending

There is some theoretical basis to assume a different impact of risk factors on offending in juvenile offenders according to neighborhood SES. Several theories have related environmental and familial risk factors such as family difficulties, parental stress, and antisocial peers to social disadvantage [7,5,8]. Therefore, these risk factors are expected to be present more often in offenders living in low SES neighborhoods. Risk factors that are less obviously related to neighborhood SES are individual risk factors, such as temperament, sensation seeking, aggression and psychiatric disorders such as attention deficit hyperactivity disorder [5,9]. Therefore, these are likely to play a role in offenders from any neighborhood. In the absence of other risk factors, individual risk factors may be expected to exert a stronger impact on juveniles in advantaged areas compared to those from disadvantaged neighborhoods, who will additionally show more environmental and familial risk factors [10,2]. Further, differences in risk factors between neighborhoods might be caused by neighborhood specific interactions [11]. For instance, attachment problems may exert a stronger impact in disadvantaged neighborhoods, because disadvantaged neighborhoods may provide these children with access to criminal opportunities and peer groups [12]. In sum, differences between neighborhoods can be expected in both the prevalence of risk factors for offending and the impact of risk factors on frequency of offending.

Until now, studies on correlates of juvenile offending by neighborhood SES have mainly focused on general population samples, using different outcome measures, such as antisocial behavior, aggression, conduct problems and delinquency. Regarding individual risk factors, both Schonberg and Shaw [10] and Beyers et al. [2] reported that these characteristics exerted a greater impact on children living in high SES neighborhoods. However, specific results have been inconsistent [1]. For instance, Lynam et al. [13] reported that impulsivity exerted a stronger influence in low SES neighborhoods, while in the study of Beyers et al. [2], ADHD had the strongest impact in high SES neighborhoods. Finally, while some studies reported the influence of deviant peers to be most pronounced in low SES neighborhoods [2], others found no area-specific relationships [14]. Overall, general population studies found mixed results regarding differences in impact of individual and peer related risk factors on level of offending according to neighborhood SES. In contrast, family characteristics have consistently been found to exert a greater impact in low SES neighborhoods [1,12,2]. Despite the fact that findings from general population based studies on the influence of neighborhood on offending carry substantial relevance, one may question the generalizability to specific offender subgroups, such as children with a first police contact.

### Ethnicity and offending

As for the role of ethnic background, some scholars state that mechanisms explaining offending are universal for all ethnic backgrounds, while others argue that these mechanisms differ by ethnic group because of cultural differences [1]. One example is the distinction between individualistic versus collectivistic cultures [15]. Many non-Western immigrants originate from collectivistic cultures in which the group is identified as the most important entity, while Western countries are generally regarded as individualistic cultures in which the individual is regarded as the most important entity [15]. It has been suggested that, because of the focus on the well being of the group, the impact of relational stress, for instance problems with peers or parents, on problem behavior such as delinquency may be higher in collectivistic cultures [16]. Further, because parental control may be seen as more legitimate in collectivistic cultures, it has been hypothesized that restrictive parental control, which is generally regarded as a risk factor for juvenile offending, does not increase offending risk among minorities [17]. However, findings on differences in impact of family factors on problem behavior have been inconsistent [18-21]. Further, it has been hypothesized that children of non-Western origin display more individual and family risk factors for offending than Western juveniles due to migration processes [for a review see [18]]. This higher level of risk factors is assumed to stem from migration stress [22], but also from the minority position in the receiving country [23]. Furthermore, children may not only suffer from their own migration stress, but also from the migration stress of their parents as stress may lead to inadequate parenting, and from the family conflicts that may arise as children tend to adjust faster to their new home country than their parents [22]. However, again, findings have been inconsistent [18]. In addition, none of the studies have focused on differences in prevalence and impact of risk factors on offending among childhood onset offenders.

### Aim of the study

Considering the above-mentioned inconsistency with regard to the relationships between offending, neighborhood SES and ethnicity, and the scarcity of research on these issues in childhood offenders, the aim of the current study was threefold. First, to describe the prevalence of risk factors in a sample of children with a first police contact below age 12 and to compare individuals from low versus moderate to high SES neighborhoods and from Dutch and non-Western origin. Second, to compare the strength of the association between risk factors and level of offending between individuals from low versus moderate to high SES neighborhoods and from Dutch and non-Western origin. Third, to study the independent association of risk factors with level of offending within each of the groups. Because only few children from non-Western origin reside in affluent neighborhoods we expected to be able to compare the following groups: *1) native Dutch offenders from moderate to high SES neighborhoods, 2) native Dutch offenders from low SES neighborhoods, and 3) offenders of non-Western origin from low SES neighborhoods*. It was hypothesized that offenders from high and low SES neighborhoods display similar prevalence rates and impact levels of individual risk factors. In addition, it was hypothesized that compared to offenders from high SES neighborhoods, offenders from low SES neighborhoods display more family and peer related risk factors and that the impact of these factors would also be higher in low SES neighborhoods. Further, it was hypothesized that individual risk factors would be the strongest independent correlates of the level of offending in offenders from high SES neighborhoods, while in offenders from low SES neighborhoods, individual, family and peer related risk factors would have an independent strong correlation with the level of offending. With regard to ethnic differences, non-Western offenders were hypothesized to display more individual and family risk factors than Dutch offenders. However, it was also hypothesized that the strength of the association between risk factors and level of offending would be similar except for parental control, which is hypothesized to have a lower impact on offending in the non-Western group. Finally, it was hypothesized that similar to low SES Dutch offenders, individual risk factors, parental and peer problems would be independent correlates of the level of offending among low SES non-Western offenders.

## Methods

### Sample

The sample consisted of 290 children who had been arrested by the police for the first time prior to age 12 because of delinquent behavior in the period July 2003-December 2005. Based on neighborhood socio-economic status (SES) and ethnicity, the following groups could be distinguished: 1) native Dutch offenders from moderate to high SES neighborhoods (n = 70), 2) native Dutch offenders from low SES neighborhoods (n = 55), and 3) offenders of non-Western origin from low SES neighborhoods (n = 105). Mean age at first arrest was 10.50 (SD = 1.16), with a range from 8 to 12. Only 13.9% was female. All offenses were of minor severity, including trespassing, shoplifting, and fighting. Almost half (45.7%) of the total group was of non-western origin. The ethnic origin of the non-Western group was distributed as follows: Moroccan (34.1%), Turkish (23.8%), Surinamese (10.3%), Dutch Caribbean (13.5%), and 18.3% of other descent.

### Procedure

Police data were obtained from local police registration systems from three different police regions covering rural and urbanized areas and different SES (*Gelderland-Midden, Utrecht, and Rotterdam-Rijnmond)*. All children who were registered for an offense by the police for the first time participated. Offending was defined as behavior that could be prosecuted or fined if displayed at the age of twelve or older (Dutch age of criminal liability). Participants' names were given by the police to the researchers when permission was granted by the parents. Next, researchers gave oral and written information about the study and obtained written informed consent from both children and parents before starting the study. The study was approved by the VU University Medical Ethics Committee and the Ministry of Justice.

A child was considered to have a non-Western background if at least one of his or her parents was born in a non-Western country [24]. Neighborhood SES was based on a five-level scale as provided by the Social and Cultural Planning Office of the Netherlands [25]. The original five levels were dichotomized into a low and moderate to high SES neighborhood grouping variable by contrasting (1)-(2) to (3)-(5)^2^.

Overall, 74.3% (N = 290) of the children referred to the researchers by the police participated in the study. Of the non-participants (n = 101), 26 parents could not be located and 75 refused participation. Non-participants did not differ from participants as to gender, age at first arrest, SES neighborhood status, or seriousness of offense resulting in arrest. Non-participants more often had a non-Western ethnic background than participants (69.6% versus 42.4%; χ^2 ^27.798(1), p < .000)^3^.

Of the 290 participants, 60 were excluded, resulting in a final sample of 230 children. Reasons for exclusion were: 1) being from non-Dutch but Western origin (n = 12), 2) being from non-Western origin but residing in affluent neighborhoods (n = 18), and 3) having verbal ability as measured by the Vocabulary subtest of the WISC-R intelligence scale [26] below 4, making comprehension of the questionnaires difficult (n = 30). Excluded children did not differ from included children as to gender, age of first arrest, or seriousness of offense leading to arrest.

### Instruments

#### Dependent variable: Level of offending

The Observed Antisocial Behavior Questionnaire (OAB: *Vragenlijst Waargenomen AntiSociaal gedrag *[27]) is based on the Self-Report of Antisocial Behavior [28] and investigates antisocial behavior over the previous half year. The child self-report and parent report versions were used to create a combined offending score (range 0 to 17). Only the items that deal with offending behavior have been included in the score. The score was based on the following 17 items: 1) stealing outside the home (5 items), 2) hitting or fighting outside the home (5 items), 3) property damage and arson (4 items), 4) rule breaking and fare dodging (2 items), and 5) weapon possession (1 item).

#### Independent variables

##### Child characteristics

The OAB Parent and Child Report was used to investigate status offending over the previous half year, by means of the following items: truancy, running away, and being expelled from school. Similarly, the OAB was used to determine substance use without parental permission. The score was based on five questions on alcohol (2 items), smoking (1 item), and drug use (2 items). Both variables were dichotomized and considered positive when scoring affirmative on at least one of the items.

Behavioral and emotional problems of the child were measured using the Strengths and Difficulties Questionnaire parent report and child report (SDQ) [29], which include the following problem scales: behavioral problems, hyperactivity, peer problems, and emotional difficulties. The SDQ is a brief behavioral screening questionnaire for 4-16 year olds [30], which can be used reliably in children from age 8 onwards [29]. The internal consistency of the scale for both parent and child report is good (α = .81 and .72) [29].

Reactive and proactive aggression were measured with the Reactive and Proactive Questionnaire (RPQ) [31,32]. The 11-item reactive subscale assesses aggression that is displayed in reaction to alleged provocation by others. The 12-item proactive subscale assesses aggression that is displayed to obtain something, i.e., not in reaction to provocation by others (e.g., "how often have you fought to show who was in charge?"). Items on both scales are answered on a three-point scale ("never", "sometimes" or "often"). The internal consistency of both subscales in the current sample was good (reactive α =.80 and proactive α =.78).

Sensation seeking was assessed using a seven-item scale asking whether or not a child would like to do exciting things (e.g., bungee jumping, exploring new places). The scale is derived from the Dutch version of the Social and Health Assessment, an assessment package used for population studies in various countries (SAHA) [33,34]. Children answer on a five-point Likert-type scale. In the current sample, the internal consistency of the scale was good (α =.70).

Affiliation with delinquent peers was assessed with a nine-item scale derived from the SAHA, asking respondents how many of their close friends ("None"; "A few"; "Some"; or "Most or all") are involved in different types of risk taking behavior such as: school, truancy, smoking cigarettes, and offending. In the current sample, the internal consistency of the scale was moderate (α =.54).

Externalizing disorders were measured with the National Institute of Mental Health (NIMH) Diagnostic Interview Schedule for Children (DISC), version IV [35]. Attention deficit hyperactivity disorder (ADHD), oppositional defiant disorder (ODD), and conduct disorder (CD) were assessed. A diagnosis of ADHD was assigned if the child met diagnostic criteria for ADD, HD or ADHD. Since ODD and CD are highly interrelated [36], and because CD at such a young age occurs infrequently and mostly in a mild form, subjects who scored either or both of these diagnoses were classified as having a DBD. For ADHD, the additional requirements were that the symptoms were present in more than one setting (school, home, outside the home) and had started prior to age 7.

##### Family and parenting characteristics

A structured checklist [37] was used to assess ethnic background, teen motherhood (below age 20), and family composition. In line with the Dutch definition, a child was considered to have a non-Western ethnic background if the child or one of his/her parents was born in a non-Western country [38].

Parental mental health problems were investigated with the Symptom Checklist SCL-90 [39,40] and four additional questions concerning psychological or psychiatric problems, alcohol abuse and drug use difficulties in the family [37]. If one or both of the parents scored affirmatively on at least one of the four questions or in the clinical range of the SCL-90, the variable was considered to be present.

Positive parenting and parental control were measured with the parenting scale as used in the SAHA. The 11-item positive parenting subscale was created by combining the parental warmth and parental involvement scale. It assesses the child's perception of parental warmth (e.g., "how often do your parents give you a hug?") and involvement (e.g., "how often do your parents ask you about your friends?"). The 8-item parental control subscale measures the child's perception of parental control by items such as "how often do your parents tell you at what time you need to come home?" Items on both scales are answered on a four-point scale ("never", "sporadically", "sometimes" or "often"). The internal consistency of the positive parenting subscale in the current sample was good (α =.74) and of the parental control scale low (α =.48).

### Statistical analyses

For statistical analysis, SPSS 13.0 was used. First, variables were described using means for continuous and percentages for categorical variables. Inter-group comparison (Dutch moderate to high SES, Dutch low SES, and non-Western low SES) was computed with χ^2 ^for categorical and analysis of variance (ANOVA) tests for continuous variables. For ANOVA, post hoc pair-wise comparisons were adjusted for multiple calculations with the Bonferroni procedure. Second, correlations between potential risk factors and level of offending were computed per offender subgroup using Pearson's r for continuous and Spearman's rho for dichotomous variables. Correlations were compared between groups using regression analyses entering the group, the independent variable and the interaction term. Finally, in order to predict level of offending within each of the three groups, regression models were constructed. Per subgroup, separate models were run for the child and family characteristics. The characteristics that uniquely contributed to these models were entered into the final model. Characteristics were entered into the model using forward selection procedures. To limit the number of variables, only variables that correlated with the dependent at a significance level of p < .10 were included. In addition, in case of the SDQ scales that were measured both in parents and in children, only the strongest correlation was entered. Due to language difficulties of the parents of the non-Western group, a substantial number of DISC based diagnoses (ADHD, DBD) was missing. Therefore, regression analyses for the low SES non-Western group were run without these variables.

## Results

### Prevalence of offending and risk factors per group

Mean numbers of offenses were respectively 1.61 (SD = 1.60, range 0-8) for the moderate to high SES Dutch offender group, 1.67 (SD = 1.80, range 0-8) for the low SES Dutch offender group, and 1.75 (SD = 1.86, range 0-8) for the low SES non-Western offender group. The distribution of the number of offenses was skewed to the left, as most children reported a low number of offenses. Therefore, in order to meet the criteria of a normal distribution, a log-transformed scale using the natural logarithm was used for further analyses. As Table 1 shows, no differences were found between the subgroups in the log transformed number of offenses. As Table 2 shows, property offenses, vandalism and rule breaking were the most commonly reported offenses. Some differences between the subgroups were found in the types of offenses that were committed. Aggression was more common in the low SES non-Western group as compared to the high SES Dutch group. Vandalism was more commonly reported by the high SES Dutch group in comparison to both low SES groups.

**Table 1:** Continuous risk variables by SES and ethnic subgroups

	moderate to high SES Dutch n = 70		low SES Dutch n = 55		low SES non-Western n = 105		All N = 230		Test	Post hoc
	Mean	SD	Mean	SD	Mean	SD	Mean	SD	F(df), p	
**Level of offending**										
Number of reported offenses (ln)	.54	.32	.51	.38	.54	.36	.53	.35	-	
**Child characteristics**										
Age onset first offense	10.92	1.22	11.10	1.21	10.75	1.12	10.88	1.17	-	
**Child report**										
Emotional problem scale	2.22	1.72	2.89	2.15	2.85	2.33	2.67	2.14	-	
Behavioral problem scale	2.77	1.70	3.23	1.61	2.69	2.01	2.84	1.84	-	
Hyperactivity scale	4.85	2.52	4.91	1.90	3.73	2.50	4.34	2.43	9.368(2), .000	b, c
Poor relationship with peers	1.86	1.48	2.94	2.02	2.48	1.89	2.41	1.85	4.675(2), .010	a, b
Proactive aggression (ln)	1.10	.71	1.28	.75	1.18	.80	1.18	.76	-	
Reactive aggression	8.56	3.60	9.75	4.06	8.91	4.48	9.01	4.15	-	
Affiliation delinquent peers (ln)	2.18	.16	2.29	.20	2.19	.22	2.21	.20	4.143(2), .017	a, c
Sensation seeking	18.92	5.70	19.06	4.85	17.00	5.87	18.06	5.65	5.974(2), .003	b, c
**Parent report**										
Emotional problem scale	2.01	2.11	2.38	2.30	2.68	2.29	2.41	2.24	-	
Behavioral problem scale	2.13	2.47	2.35	2.07	2.46	2.35	2.33	2.32	-	
Hyperactivity scale	4.36	3.24	4.85	2.89	3.80	2.51	4.24	2.87	4.733(2), .010	c
Poor relationship with peers	1.47	1.80	2.04	2.15	2.24	1.79	1.94	1.91	2.558(2), .080	

**Family characteristics**										
Low positive parenting	7.43	4.24	8.40	4.32	7.12	4.54	7.52	4.41	-	
Low parental control	4.80	2.88	5.36	3.57	3.89	3.09	4.51	3.19	4.519(2), .012	b

Note. (ln) Transformed using natural logarithm to meet criteria of normal distribution

a. post-hoc difference between moderate to high SES Dutch and low SES Dutch

b. post-hoc difference between moderate to high SES Dutch and low SES non-Western

c. post-hoc difference between low SES Dutch and low SES non-Western.

**Table 2:** Dichotomous risk variables by SES and ethnic subgroups

	moderate to high SES Dutch n = 70	low SES Dutch n = 55	low SES non-Western n = 105	All N = 230	Test	Post hoc
	%	%	%	%	**χ**^**2**^**(df), p**	
**Offense type self reported offending**						
Aggression	5.8	10.9	20.9	13.9	8.687(2), .013	b
Property	27.1	32.7	32.4	30.9	-	
Vandalism	50.0	30.9	24.8	33.9	12.228(2), .002	a, b
Rule breaking	27.3	31.0	37.1	32.6	-	
Weapon possession	2.9	5.5	3.8	3.9	-	

**Child characteristics**						
Gender (% girl)	5.7	18.2	17.1	13.9	5.680(2), .058	a, b
Status offense	7.1	10.9	24.8	16.1	11.094(2), .004	b, c
Substance use	18.6	23.6	15.2	18.3	-	
Externalizing disorder	27.2	41.8	30.2	32.3		
ADHD	22.9	23.6	24.7	23.7	-	
DBD	15.7	30.9	20.8	21.8	-	
*ADHD+DBD*^*1*^	*11.4*	*12.7*	*15.3*	*13.2*	-	
**Family characteristics**						
Teen mother	2.9	5.5	28.0	14.7	25.474(2), .000	b, c
Not both biological parents in home	25.7	47.3	50.5	42.1	11.285(2), .004	a, b
Parental mental health problems	28.6	41.8	32.0	33.3	-	

Note.

a. post-hoc difference between moderate to high SES Dutch and low SES Dutch

b. post-hoc difference between moderate to high SES Dutch and low SES non-Western

c. post-hoc difference between low SES Dutch and low SES non-Western

1. children in the ADHD+DBD group are also represented in the ADHD and DBD groups above.

Given the young age of these offenders, most risk factors were highly prevalent in all three groups; e.g., status offenses (16.1%) and substance use (18.3%). In addition, almost one third of the children met the criteria for DBD or ADHD, while almost half of those children (13.2%) met the criteria for both DBD and ADHD (Tables 1 and 2). Furthermore, one third of the children had a parent with mental health problems and 42.1% were not living with both their biological parents.

Tables 1 and 2 show differences in prevalence of risk factors between groups. First, a number of characteristics was more prevalent in children from low SES neighborhoods (regardless of ethnic background) than in offenders from moderate to high SES neighborhoods. Offenders from low SES neighborhoods were more often female, reported significantly poorer relationships with peers, and more often came from broken families. In addition, children from the low SES Dutch offender group more often affiliated with delinquent peers than children from the moderate to high SES Dutch offender group.

Compared to the low SES non-Western offender group (Tables 1 and 2), both Dutch groups were higher in hyperactivity and sensation seeking. Further, Dutch offenders from low SES neighborhoods reported more delinquent peer affiliation than non-Western children from low SES neighborhoods. On the other hand, non-Western children reported more status offenses than Dutch children from low and moderate to high SES neighborhoods. Finally, both Dutch groups less often had a mother who was a teenager at birth and reported higher levels of low parental control.

### Correlations between offending and risk factors

Tables 3 and 4 provide correlations between risk factors and level of offending for each of the three groups. First, the common correlations will be described, followed by a description of differences between groups. In all three groups, behavioral problems and reactive aggression as reported by the child were associated with higher levels of offending, as were status offenses and parent reports of DBD, ADHD, behavioral problems, and hyperactivity.

**Table 3:** Parametric correlations of risk variables with level of offending per group

	moderate/high SES Dutch n = 70	low SES Dutch n = 55	low SES non-Western n = 105	All N = 230	Sign difference between correlation
	r	r	r	r	
**Child characteristics**					
Age onset first offense	.021	.132	-.122	-.004	-
**Child report**					
Emotional problem scale	.006	.046	.062	.040	-
Behavioral problem scale	.344***	.340***	.309***	.315***	ns
Hyperactivity scale	.146	.196	.377***	.259***	b*
Poor relationship with peers	-.038	.021	.110	.040	ns
Reactive aggression	.268**	.272**	.257***	.255***	ns
Proactive aggression (ln)	.143	.381***	.320***	.286***	ns
At risk peer affiliation (ln)	.029	.201	.277**	.188***	ns
Sensation seeking	.247**	.211	.405***	.308***	ns
**Parent report**					
Emotional problem scale	.102	.314**	.274***	.234***	ns
Behavioral problem scale	.370***	.463***	.440***	.417***	ns
Hyperactivity scale	.319***	.340**	.341***	.324***	ns
Poor relationship with peers	.176	.250*	.389***	.278***	b*

**Family characteristics**					
Low positive parenting	.310**	.125	.207**	.209'''	ns
Low parental control	.045	.000	.150	.074	-

Note. * p < .1

** p < .05

*** p < .01

a. post-hoc difference between moderate to high SES Dutch and low SES Dutch

b. post-hoc difference between moderate to high SES Dutch and low SES non-Western

c. post-hoc difference between low SES Dutch and low SES non-Western.

**Table 4:** Non-parametric correlations of risk variables with level of offending per group

	Dutch Moderate/high SES n = 70	Dutch low SES n = 55	Non-Western low SES n = 105	All N = 230	Sign difference between correlation
	Rho	Rho	Rho	Rho	
**Child characteristics**					
Gender (% girl)	-.040	.057	.031	.030	-
Status offenses	.288**	.446***	.253***	.298***	c*
Substance use	.198	.060	.367***	.230***	c*
ADHD	.394***	.358***	.374***	.377***	ns
DBD	.301***	.401***	.459***	.387***	ns

**family characteristics**					
Teen mother	-.055	.267**	.099	.095	ns
Not both biological parents in home	.272**	.220	.255***	.247***	ns
Parental mental health problems	.101	.269**	.306***	.239***	b*

Note. * p < .1

** p < .05

*** p < .01

a. post-hoc difference between moderate to high SES Dutch and low SES Dutch

b. post-hoc difference between moderate to high SES Dutch and low SES non-Western

c. post-hoc difference between low SES Dutch and low SES non-Western.

Some correlations were found in some but not all subgroups. However, only few statistical differences in the strength of correlation between risk factors and level of offending were found between the three groups. Further, those differences that were found, were only found at the trend level. Regarding neighborhood specific correlations, proactive aggression, emotional problems and poor relationships with peers as reported by the parent and parental mental health problems were only associated with higher levels of offending in both low SES groups. The strength of the correlation of the latter three differed significantly between the low SES non-Western and the high SES Dutch group.

Further, some ethnic specific correlations were found. Substance use, at risk peer affiliation and hyperactivity as reported by the parent only positively correlated with level of offending in the non-Western group. The difference in correlation of substance use with offending was significant between the low SES Dutch and low SES non-Western group. Finally, low positive parenting, sensation seeking and not having both biological parents at home were associated with higher levels of offending in the high SES Dutch and the low SES non-Western group, while teen motherhood was only associated with higher levels of offending in the low SES Dutch group. However, none of these correlations differed significantly between the three groups.

### Explaining variance in level of offending per subgroup

Table 5 shows risk factors that contributed independently to the variance in level of offending for each group separately. In the Dutch moderate to high SES group, ADHD and behavioral difficulties as reported by the parent explained 22.7% of the variance in the individual risk factor model. Low positive parenting and not living with both biological parents predicted 14.7% of the variance when entered in the family model, but no longer uniquely explained variance in offending in the combined model. The combined model was the same model as the individual risk factor model.

**Table 5:** Regression analyses per group

		N	Variables	beta	Sign (p)	**R**^**2**^	Anova F(df), p
Moderate/High SES Dutch^1^	Child characteristics^4^	64	ADHD (p) Behavior problem (p)	.302 .261	.019 .042	.231	9.139(2), .000
	Family characteristics	66	Low positive parenting	.310	.023	.092	6.804(2), .011
	Combined model	64	ADHD (p) Behavior problem (p)	.302 .261	.019 .042	.231	9.139(2), .000

Low SES Dutch^2^	Child characteristics^5^	53	Behavior problem (p) Proactive aggression (c)	.396 .273	.002 .032	.290	10.229(2), .000
	Family characteristics		No variables entered				
	Combined model	53	Behavior problem (p) Proactive aggression (c)	.396 .273	.002 .032	.290	10.229(2), .000

Low SES non-Western (excl. diagnoses)^3^	Child characteristics^6^	94	Sensation seeking Behavior problem (p) Substance use Poor relationship with peers	.236 .224 .254 .213	.012 .023 .005 .025	.356	12.001(4), .000
	Family characteristics	100	Mental health problems parent Not both parents in the home	.298 .250	.002 .009	.160	9.235(2), .000
	Combined model	93	Behavior problem (p) Poor relationship with peers (p) Substance use Parental mental health problems Sensation seeking (c)	.198 .207 .244 .239 .224	.040 .025 .005 .006 .011	.412	12.207(5), .000

Note. (p) parent report

(c) child report

Given the limited sample sizes of both Dutch subsamples, only the strongest correlations were entered in the regression analyses up to a maximum of 5. Collinearity proved not to be a problem. However, to limit overlap between constructs, if both child and parent report on the same SDQ scale were correlated, the strongest one was entered. In both Dutch groups, if both a behavior problem scale and a DBD diagnosis or both a hyperactivity scale and an ADHD diagnosis were correlated, the strongest one was entered.

1. If the analyses were run excluding the psychiatric diagnoses ADHD en DBD, only behavior problems entered the model.

2. Running the regression analyses without the psychiatric diagnoses ADHD and DBD produced the same model.

3. The regression analyses including the psychiatric diagnoses ADHD and DBD produced the same model but in a smaller sample.

4. ADHD, parent report behavioral problems, status offenses, reactive aggression, sensation seeking and proactive aggression were entered in the model.

5. Parent report emotional problems, parent report behavioral problems, proactive aggression, ADHD en status offenses were entered in the model.

6. Sensation seeking, parent report behavioral problems, substance use, parent report poor relationship with peers and child report hyperactivity were entered.

In the low SES Dutch offender group, 29.0% could be explained by parent reports of behavioral problems and child reports of proactive aggression. No variables entered the family model. As a result, the combined model was the same as the individual risk factor model.

Finally, in the low SES non-Western offender group more than a third of the variance (35.6%) could be explained by substance use, sensation seeking, behavioral problems, and problems in the relationship with peers of the child. Although parental mental health problems as well as not living with both biological parents uniquely contributed to the family model (16.0% explained variance), only parental mental health problems entered the combined model (41.2% overall explained variance).

## Discussion

The current study focused on the prevalence of risk factors and the correlation of these factors with levels of self and parent reported offending in childhood arrestees from different neighborhoods and ethnic backgrounds. Overall, high rates of risk factors were found in each of the groups, particularly family difficulties and externalizing disorders. Contrary to our hypothesis, few differences were found in the prevalence of individual and family risk factors between individuals from disadvantaged versus affluent neighborhoods. In line with our hypothesis, peer related risk factors were found to be more common in the low SES groups than in the moderate to high SES group. Further, few differences were found between neighborhoods in the strength of the association between risk factors and level of offending. As regards ethnic differences within low SES neighborhoods, in line with our hypothesis non-Western children had more family risk factors. Further, contrary to our hypothesis, substance use, self-reported hyperactivity, and sensation seeking stood out as relatively strong correlates of offending in de low SES non-Western group, while status offences was a unique correlate in the low SES Dutch group. Finally, in the multivariate models, few and only behavior related individual risk factors independently correlated with frequency of offending across neighborhoods for the Dutch groups: ADHD and behavioral problems in the moderate to high SES Dutch group, and behavioral problems and proactive aggression in the low SES Dutch group. Interestingly, in the low SES non-Western group, not only individual but also parental and peer factors correlated uniquely with level of offending.

As we examined a group of first-time police arrestees under the age of 12, the high levels of externalizing psychiatric disorders and family difficulties may be considered alarming. For instance, almost one third met the criteria for CD, ODD, and/or ADHD, which is high compared to the eight percent of externalizing disorders found in the Dutch general population [41]. Moreover, 13.2% met the criteria for both DBD and ADHD, which has been found to increase the risk of antisocial behavior in general and future offending in specific [42]. A first police encounter may therefore offer an opportunity to identify a high-risk group, which may well be difficult to detect in the general population. On the other hand, although risk factors were high when compared to the general population, still a large proportion of participating children did not show an increased level of risk factors. This may also be important for prediction and intervention purposes. If the less troubled children are the ones who will develop well and abstain from further delinquency, methods of early detection are essential. First, to avoid over-intervention in the relatively large group that is not showing any problems. Second, to use scarce financial means for the treatment of those most in need. While children who show many risk factors are likely to need intensive attention, children who display few risk factors may still benefit from less intensive intervention as these few risk factors may serve as stepping stones to more severe problems if left unattended. Therefore, it may be most appropriate to use a stepped care model aimed at both the parent and the child, ranging from less intensive interventions to prevent low risk children from becoming at risk to intensive interventions aimed at avoiding persistence in high risk children.

There may be several explanations for the relative lack of differences in the prevalence of individual risk factors. First, early police arrestees are likely to be a particular selection of the normal population similar to the early onset offenders as described by Moffitt [5], a group facing substantial individual problems, regardless of neighborhood status. Second, neighborhood SES reflects an average of the SES level of the households residing in that area. However, even within moderate to high SES neighborhoods, children from relative low SES families may be the ones who get arrested. Hence, this group may resemble low SES arrestees quite closely with respect to familial characteristics. However, parental mental health problems and teen motherhood demonstrated a stronger correlate to level of offending in Dutch children residing in low as compared to high SES neighborhoods. This could indicate that although prevalence rates may be similar, parents in low SES neighborhoods receive less support and/or treatment for their problems. As a result the problems may have a stronger impact on the behavior of the child. Finally, peer related risk factors were more prevalent among Dutch offenders from low as compared to moderate to high SES neighborhoods. This could become of importance when these children grow older and start to spend more time outside the home in the presence of their peers. As a result, the interaction with antisocial peers may become a stronger risk factor for the persistence of offending in adolescence among childhood onset offenders from low SES neighborhoods.

Low SES non-Western offenders reported fewer peer related risk factors compared to low SES Dutch children. However, the correlation between at risk peer affiliation and offending was similar while the correlation with poor relationships with peers was stronger for the non-Western group. This might indicate that rejection by others may be a particularly important risk factor for ethnic minorities. Future research should go further into this difference between prevalence and impact of peer related risk factors between ethnic groups. In contrast, although teen motherhood was more common within the low SES non-Western group teen motherhood was only correlated with offending in the low SES Dutch group. The higher levels of teen motherhood in non-Western minorities reflect similar differences between ethnic groups in the general population [43]. Contrary to our hypothesis, low SES non-Western offenders did not report more individual and parental risk factors. However, as minority groups have been described as prone to socially desirable responding concerning their behavior [44], this may have influenced the findings in the low SES non-Western group. Therefore, further research should investigate whether these findings also hold when different informants or observational measures are used.

In both Dutch groups, few risk factors independently correlated with level of offending. Contrary to our hypothesis, family or parenting characteristics did not correlate independently with level of offending in the low SES Dutch offender group. The finding that only individual risk factors independently predicted level of offending could partly be due to the larger number of individual as compared to parental and peer risk factors that were studied. Among the individual risk factors, only those reflecting externalizing behavior independently predicted level of offending. The finding that differences in level of offending are best explained by differences in the level of other problem behaviors of the children may not come as a surprise. However, when only family characteristics were taken into account it proved difficult to distinguish between children in terms of reported offending. Furthermore, the overall low correlations between risk factors and level of offending stresses the difficulty of differentiating serious from non-serious offenders on the basis of a single characteristic. Given the absence of an official offense history in these children, the assessment of self-reported behavioral difficulties seems of particular clinical relevance in this group. However, this would require a different approach by the police, who are now likely to rely solely on official offending data. Given the importance of obtaining information useful for detecting high-risk children, the issue of an independent psychodiagnostic assessment following a first police contact needs further consideration.

In line with the hypothesis, family and peer related risk factors as well as individual factors uniquely correlated with level of offending in the low SES non-Western group. As this was not so in the Dutch groups, this argues for differentiating between ethnic origins when studying correlates of offending. In addition, it may be essential to study the broader environment of the child when assessing offending risk in this group. The association between offending level and parental mental health problems in the non-Western group is also of interest. Minorities are known to receive less specialized help for their mental health problems [45], which may interfere with quality of parenting and result in less positive parenting styles.

### Limitations

A number of shortcomings must be considered when interpreting the results of the present study. First, because the study had a cross sectional design, no inferences can be made regarding causality. Second, the non-Western group was heterogeneous, representing different cultural values and beliefs. Third, collecting information was especially difficult in the non-Western sample. Many parents had problems answering questions due to language difficulties, while cultural differences may have led to a different interpretation of questions. Finally, due to relatively low subgroup sample sizes we were unable to test for interactions between potential risk factors or between subgroup membership and risk factors. Therefore, we were not able to make firm inferences about differences in the impact of risk factors on offending between the subgroups.

## Conclusions

Notwithstanding these limitations, results from this study demonstrate that children with an early police encounter are a high-risk group, many of whom are in need of mental health and family treatment regardless of their background. Therefore interventions should be delivered according to a stepped care model and should be aimed at individual, family and peer related risk factors regardless of the origin of the child. Few neighborhood differences have been found in the impact of individual and parental risk factors on offending. However, the predictive validity of these risk factors still must be investigated prospectively. Some differences were found between ethnic minorities and the Dutch group, particularly in the independent correlation of not only individual but also family and peer risk factors on level of offending. This implies that a broader context should be considered when screening for at risk non-Western children. However, before firm conclusions can be made, different ethnic minorities should be studied separately and potential cultural and immigration dependent risk factors should be included.

## Competing interests

The authors declare that they have no competing interests.

## Authors' contributions

LD carried out the study and drafted the manuscript. RV supervised the study, participated in its design and helped to draft the manuscript. TD supervised the study, participated in its design and helped to draft the manuscript. CG carried out the study with LD. All authors read and approved the final manuscript.
